# Supplementary material for: Does amiodarone impact on apixaban levels? The effect of amiodarone on apixaban level among Thai patients with non-valvular Atrial Fibrillation
Source: PLoS One. 2024 Jan 19;19(1):e0295511. doi: 10.1371/journal.pone.0295511 (PMC10798445; doi:10.1371/journal.pone.0295511)
Supplement: Attachment — Submitted filename: Responses to reviewer-PONE-D-23-05377.doc [file pone.0295511.s001.doc]

**Responses to comments for Manuscript ID: PONE-D-23-05377 [Major Revisions] entitled “Does amiodarone impact on apixaban levels? A significant of trough concentration apixaban among patients with non-valvular atrial fibrillation”**

Please include the following items when submitting your revised manuscript:

- A rebuttal letter that responds to each point raised by the academic editor and reviewer(s). You should upload this letter as a separate file labeled 'Response to Reviewers'.
- A marked-up copy of your manuscript that highlights changes made to the original version. You should upload this as a separate file labeled 'Revised Manuscript with Track Changes'.
- An unmarked version of your revised paper without tracked changes. You should upload this as a separate file labeled 'Manuscript'.

**Comments to the Author**

**Comment # 1** Is the manuscript technically sound, and do the data support the conclusions?

The manuscript must describe a technically sound piece of scientific research with data that supports the conclusions. Experiments must have been conducted rigorously, with appropriate controls, replication, and sample sizes. The conclusions must be drawn appropriately based on the data presented.

**Reviewer #1:** Partly

**Reviewer #2**: Partly

**Response:**

Thank you for your suggestion. We provided this sentence “To the best of our knowledge, amiodarone affected apixaban plasma at the peak and trough levels, especially trough levels were statistically significantly increased. Based on the results of this study, no clinically significant bleeding events occurred in the comparison of the apixaban-monotherapy and apixaban-with-amiodarone groups. However, this study had a small number of patients, so close monitoring of bleeding symptoms in patients who used concomitant apixaban with amiodarone were necessary.” in conclusion section, page 12.

**Comment # 2** Has the statistical analysis been performed appropriately and rigorously?

**Reviewer #1: No**

**Reviewer #2: Yes**

**Response:**

Thank you for your valuable comments. We have already provided the statistical analysis section as follows: statistical analysis was performed using the Statistical Package for the Social Sciences (SPSS) statistics version 27.0. (IBM Corp, Armonk, NY). All variables were analyzed using descriptive statistics to determine the frequencies with percentages for categorical variables, while continuous variables were expressed in mean ± standard deviations (SDs) or median with interquartile range (IQR). The apixaban plasma levels were reported in median [5th – 95th] percentiles based on the 2021 European Heart Rhythm Association Practical Guide on the Use of Non-Vitamin K Antagonist Oral Anticoagulants in Patients with Atrial Fibrillation. All patients were included and classified into 2 groups. The propensity scores were used to balance covariates in baseline characteristics such as age, body weight, SCr and CrCl. The Mann-Whitney U test was used to compare differences of apixaban level and anti-FXa activity between control and apixaban with amiodarone groups. Two-tailed p-value<0.05 was considered statistically significant.” as reviewer’s comments.

**Comment # 3** Have the authors made all data underlying the findings in their manuscript fully available?

The PLOS Data policy requires authors to make all data underlying the findings described in their manuscript fully available without restriction, with rare exception (please refer to the Data Availability Statement in the manuscript PDF file). The data should be provided as part of the manuscript or its supporting information, or deposited into a public repository. For example, in addition to summary statistics, the data points behind means, medians and variance measures should be available. If there are restrictions on publicly sharing data—e.g. participant privacy or use of data from a third party—those must be specified.

**Reviewer #1:** Yes

**Reviewer #2:** No

**Response:**

Thank you for your valuable suggestion. We provided the sentence “The datasets used and/or analyzed during the current study are available from the corresponding author on reasonable request.” in Availability of Data, on page 12.

**Comment # 4** Is the manuscript presented in an intelligible fashion and written in standard English?

PLOS ONE does not copyedit accepted manuscripts, so the language in submitted articles must be clear, correct, and unambiguous. Any typographical or grammatical errors should be corrected at revision, so please note any specific errors here.

**Reviewer #1:** No

**Reviewer #2**: No

**Response:**

This revised manuscript has been proofread and edited by a native English speaker specializing in Medical English.

**Comment # 5 Review Comments to the Author**

Please use the space provided to explain your answers to the questions above. You may also include additional comments for the author, including concerns about dual publication, research ethics, or publication ethics. (Please upload your review as an attachment if it exceeds 20,000 characters)

**Reviewer #1:**

Limcharoen et al included 33 NVAF patients who were receiving apixaban and compared the medication consternation and bleeding risk between those who received concurrent amiodarone and those who did not. The found patients with concurrent amiodarone use had significantly higher trough concentration of apixaban, but was not associated with bleeding risk. There are a few issues to address/clarify:

1. The study design was confusing. The section Study participants suggests the patients were already receiving apixaban with or without amiodarone before enrollment since adherence to the use of these medications within 7 days before enrollment were examined (Page 3), and the patients were further retrospectively categorized according to use of amiodarone. However, in the result section (Page 5), the authors stated “We classified the patients into two groups by propensity score matching (1: 2): 11 patients were treated with apixaban-with- amiodarone, and 22 were subjected to apixaban-monotherapy (control group).” This seems to suggest the assignment of amiodarone was actually conducted by the authors. If this is the case, I wonder whether it is ethical to prescribe amiodarone without a clear (prespecified) indication. More details about this should be provided. Also, for a prospective interventional study, why not consider a random assignment?

**Response:**

Thank you for your valuable comments. We revised the phrase to “All patients were included, who were already receiving apixaban with or without amiodarone before enrollment since adherence to the use of these medications within 7 days before enrollment were examined by telephone and pill count method. After that patients were categorized into two groups as follows: the included patients were categorized into two groups: apixaban-monotherapy (control group) and apixaban-with-amiodarone groups.” page 3. In addition, we added the explanation in this phrase “During the study period, 109 NVAF patients were identified from the hospital’s electronic database. *All those patients were already receiving apixaban with or without amiodarone before enrollment.* A total of 69.7% of the patients were excluded because of enrollment denials in 62 patients (the patients denied to sign informed consent because they denied venipuncture and more than half of patients received medication delivery), CrCl less than 15 mL/min or required hemodialysis in 12 patients, and inability to conduct self-care in 2 patients. Therefore, a total of 33 patients were enrolled. We classified the patients into two groups by propensity score matching (1:2): 11 patients were treated with apixaban-with-amiodarone, and 22 received apixaban-monotherapy.” in results section (page 5). We did not consider a random assignment because this study had a small number of patients.

2. In the manuscript it was stated that the study was conducted from January 2021; however, according to the TCTR registry (TCTR20210311005), the first enrollment was actually 30 December 2020. The authors should keep the manuscript consistent with the actual enrollment. I also noticed that study was retrospectively registered, which should be mentioned in the manuscript.

**Response:**

We apologize for this and thank you for your suggestion. We corrected this sentence “This study was a prospective, observational, and single-center research which was conducted from January 2021 to January 2022 among patients receiving apixaban at Phramongkutklao Hospital, a tertiary care hospital located in the center of Bangkok, Thailand. The study protocol was approved by the institutional review board of the Royal Thai Army Medical Department and Phramongkutklao Hospital (Approval No. S054h/63) and registered in the Thai Clinical Trials Registry. The identification number is TCTR20210311005.” to “This study was a prospective, observational, and single-center research which was conducted from *October 2020 to September 2022* among patients receiving apixaban at Phramongkutklao Hospital, a tertiary care hospital located in the center of Bangkok, Thailand. The study protocol was approved by the institutional review board of the Royal Thai Army Medical Department and Phramongkutklao Hospital (Approval No. S054h/63) and registered in Thai Clinical Trials Registry. The identification number is TCTR20210311005 *and this study was retrospectively registered.”*

3. Details about the propensity score matching should be described. If the two groups were assigned by propensity scores, it would not be surprised to see “most baseline 190 characteristics were similar between the groups”.

**Response:**

Thank you for your suggestion. We described about propensity score matching in this sentence “All patients were included and classified into 2 groups. The propensity scores were used to balance covariates in baseline characteristics such as age, body weight, SCr and CrCl.” in the statistical section (page 5, line 82).

4. For the investigated clinical outcomes, how were they identified? How long were the patients followed for? These details should also be clearly described.

**Response:**

Thank you for your comment. In this study, after the patients had been identified electronic chart reviews were manually reviewed, and relevant data were extracted and transferred into the case record form for all cases. Patients were followed until the first occurrence of bleeding outcomes of interest or until 1 year. We explained that “After the blood samples were reported manual electronic chart reviews were performed and all patients were followed until the first occurrence of bleeding outcome of interest or until 1 year.” in the outcome of interest section, page 4 (line 62).

5. In the result section, it was mentioned “During the 1-year study period following up…” Since the study was conducted between January 2021 and January 2022, would this mean the majority of the included patients were actually followed for less than one year? If this is the case, censoring should be taken into account for the clinical outcome analysis. In the registered protocol, the anticipated last enrollment would be June 30 2022, and the completion date of the study would be December 2022. I wonder what made the authors terminate the study earlier.

**Response:**

We apologize for this. This study was conducted to collect blood samples between January 2021 to January 2022, because this study was limited by COVID*-*19 epidemic situation. In the results section, it was mentioned “During the 1-year study period”, we meant that patients were followed up for the bleeding outcomes by electronic chart reviews for 1 year after blood samples were collected from patients. Censoring of data was conducted when the bleeding outcome occurred or at 1 year. Therefore, we amended the study protocol for extended duration of bleeding outcome following up to December 2022. This sentence “This study was a prospective, observational, and single-center research and conducted from *January 2021 to January 2022*….” has been changed to “This study was a prospective, observational, and single-center research which was conducted from *October 2020 to September 2022* ….” in the Materials and methods section, page 3 (line 98).

6. The planned sample size was 130 according to the TCTR registry (TCTR20210311005), while only 33 were included only (from 109 patients). How was the sample size estimated? What caused the change?

**Response:**

We apologize for this. Therefore, we have changed “TCTR20210311*005*” to “TCTR20201005*003*”. The planned sample size was 50 patients according to the TCTR registry (TCTR20201005003). We estimated the sample size according to Charles Frost, et al., (Br J Clin Pharmacol., 2013;76(5):776–786), so this study estimated the sample size was 48 patients. Because this study was limited by COVID-19 epidemic situation, so the sample sized was less than numbers estimated (most patients denied the informed consent).

7. Relevant studies about potential impact of amiodarone use on bleeding risk among AF patients receiving apixaban were not well discussed. In a recent large-scale study (PMID 37216662), concurrent use of amiodarone was found to be associated with increased risk of bleeding-related hospitalizations. I think this further suggests the current study might be underpowered to observe any difference in bleeding outcomes.

**Response:**

Thank you for your comment and we agree with your comment. It is a limitation of our study that we discussed “First, this work used a small sample size and did not evaluate the relationship between bleeding outcomes and apixaban plasma levels, including the anti-FXa activity, in the apixaban-with-amiodarone group. Therefore, multicenter or larger sample sizes should be used in future studies.” page 12 (lines 112).

8. Apixaban plasma levels were presented as median (5th-95th percentiles). I wonder why the authors preferred this, instead of median with IQR (i.e., 25-75th percentiles).

**Response:**

Thank you for valuable comments. We used median [5th – 95th] percentiles based on apixaban plasma levels that there were reported to range in “2021 European Heart Rhythm Association Practical Guide on the Use of Non-Vitamin K Antagonist Oral Anticoagulants in Patients with Atrial Fibrillation”. Therefore, we added the sentence “The apixaban plasma levels were reported in median [5th – 95th] percentiles *based on the 2021 European Heart Rhythm Association Practical Guide on the Use of Non-Vitamin K Antagonist Oral Anticoagulants in Patients with Atrial Fibrillation.*” in the statistical analysis part, on page 5 (line 74).

9. For analysis of the bleeding outcomes, occurrence of the bleeding outcomes was not directly compared between the two groups (i.e., apixaban without amiodarone versus apixaban with amiodarone). Instead, the authors compared amiodarone use in patients with bleeding events to those without. Such an analysis seems rather rare to see, and I think here the comparison lost the benefit of confounding control by propensity score matching.

**Response:**

Thank you for your valuable comments. We provided this sentence “In addition, the finding of this study showed a profound bleeding outcome difference between apixaban-with-amiodarone and control group (2 events (18.18%) and 5 events (22.73%), respectively; p = 0.763). However, no statistically significant difference was observed between apixaban-with-amiodarone and control groups” in results section, on page 6 (lines 42-46).

10. Many analyses (particularly about the bleeding outcomes) were not mentioned at all in the section Statistical analysis.

**Response:**

Thank you for your suggestion. We mentioned that “Bleeding outcomes were expressed as percentage while Chi-square test were used to identify statistical differences” on page 5 in the statistical analysis section (line 80) following your suggestion.

11. “Notably, the peak and trough of apixaban plasma level in the apixaban-with-amiodarone group was higher than that in the apixaban-monotherapy group and statistically significant.” This statement was incorrect, as the authors found no significant difference in apixaban Cpeak. This issue applies to the conclusion “Amiodarone affected the peaks and troughs of apixaban plasma levels”.

**Response:**

We apologize for this and thank you for your comments. We corrected this sentence “Notably, the peak and trough of apixaban plasma levels in the apixaban-with-amiodarone group was higher than that in the apixaban-monotherapy group and statistically significant.” This statement was incorrect, as the authors found no significant difference in apixaban Cpeak to “The principal findings of this study indicated the peak and trough of apixaban plasma level in the apixaban-with-amiodarone group was higher than that in the apixaban-monotherapy group and statistically significant in Ctrough.” in the discussion part, on page 10 (line 102).

12. In the introduction, the authors stated “… but no pharmacokinetic data are available for the apixaban group” However, in the discussion, they gave many examples (e.g., reference 19, 15, 20) about existing studies that investigated pharmacokinetic data of apixaban.

**Response:**

Thank you for your suggestion. Based on this sentence “Clinical practice guidelines showed the effect of amiodarone on the increase in area under the curve (AUC) of dabigatran, edoxaban, and rivaroxaban (12%–60%, 40%, and minor effect, respectively), but *no pharmacokinetic data are available for the apixaban group* [14]” that we mentioned in pharmacokinetic data especially as guideline reported. As your suggestion, we changed and added the reference in this sentence “Theoretically, the pharmacokinetic interaction between apixaban and amiodarone may result in increased apixaban plasma levels and bleeding [13]. Clinical practice guidelines reported the effect of amiodarone on the increase in area under the curve (AUC) of dabigatran, edoxaban and rivaroxaban (12%–60%, 40% and minor effect, respectively), *but limited pharmacokinetic data are available for the apixaban group* [14]. Therefore, the interaction between amiodarone and apixaban has been reported in caution. *The data finding from the previous observational studies is controversial, some studies reported that clinical outcomes of amiodarone combined with apixaban did not increase the risk of stroke and major bleeding compared with warfarin while other studies reported the apixaban levels only [15-19]*.*”* in the introduction section, on page 2 (lines 75-76).

13. **Minor points:**
    1) Title: There is a grammatical error in the title “A significant of …”. Did the authors mean “significance of …”?

**Response:**

Thank you for your suggestion. Following this comment and reviewer 2’s comment, we changed the title to “Does amiodarone impact on apixaban levels? The effect of amiodarone on apixaban level among Thai patients with non-valvular AF.

2) Keywords: Consider to add Amiodarone into the keywords.

**Response:**

Thank you for your valuable comments. We added “amiodarone” into the keywords.

3) Ethics Statement: There is a grammatical error “All eligible provided …”

**Response:**

We apologized for grammatical error. We corrected this sentence to “Patients were eligible for recruitment if they were ≥18 years old, had received apixaban for at least 7 days, diagnosed with NVAF, and provided informed consent.” in the study participants section, on page 3.

4) Abstract: Avoid using causal wordings in the conclusion.

**Response:**

We apologize for this and we revised and carefully used wording in abstract part.

5) P value was missing in the last sentence of the results in the abstract. There seems also a typo “in the couple”.

**Response:**

We apologize for this and we changed this sentence “Likewise, C peak exhibited no difference in the both (148.69 [141.92–283.45] µg/L) and bleeding (187.74 [1162.92–238.80] µg/L) groups” to “Likewise, Cpeak showed no difference in both non-bleeding (148.69 [141.92–283.45] µg/L) and bleeding (187.74 [1162.92–238.80] µg/L) groups, p=449”, on page 1 (line 37).

6) The reference 4 did not fully support the statement “The three major goals of treatment are …”.

**Response:**

We apologized for that mistake and we changed the reference 4 to “Hindricks G, Potpara T, Dagres N, Arbelo E, Bax JJ, Blomström-Lundqvist C, et al. 2020 ESC Guidelines for the diagnosis and management of atrial fibrillation developed in collaboration with the European Association for Cardio-Thoracic Surgery (EACTS). Eur Heart J. 2021;42(5):373-498.”

7) Proofreading is needed about the English language and use of punctuations.

**Response:**

This revised manuscript has been proofread and edited by a native English speaker specializing in Medical English.

**Reviewer # 2:**

In the study the authors compared the apixaban levels and bleeding outcomes between apixaban monotherapy and apixaban with amiodarone therapy groups. The research question is interesting and relevant. Furthermore, the prospective design of the study improves the quality. However, I have some concerns and questions. Most importantly, the sample size of only 7 bleeds is too small to draw statistical conclusions on the association between bleeding outcomes and the concomitant use of amiodarone.

**1 General:**

\# The English language in the paper could be improved. Importantly, the title of the paper should be altered to improve the reading quality. Furthermore, several sentences are not correct. Please check your full paper on English language.

**Response:**

We apologized for this that we sent this MS for proofreading again. This revised manuscript has been proofread and edited by a native English speaker specializing in Medical English.

\# For instance, page 2 line 75-76 could be changed to ‘Theoretically, the pharmacokinetic interaction between apixaban and amiodaron may result in increased apixaban plasma levels and bleeding.’

**Response:**

Thank you for your suggestion. We changed this sentence “The pharmacokinetic of amiodarone and apixaban can increase the apixaban plasma levels and bleeding” to “Theoretically, the pharmacokinetic interaction between apixaban and amiodarone may result in increased apixaban plasma levels and bleeding.” as your suggestion, on page 2 line 75-76.

**2 Abstract:**

Page 1, line 37. please clarify ‘couple’ group.

**Response:**

We apologized for this that we changed the sentence to “Likewise, Cpeak showed no difference in the non-bleeding (148.69 [141.92–283.45] µg/L) and bleeding (187.74 [1162.92–238.80] µg/L) groups, p=449.” in abstract section, page 1 (line 37).

**3 Introduction:**

\# Page 2, line 79. I regard plasma peak/trough concentrations also as pharmacokinetic data. So your statement that no pharmacokinetic data are available for the apixaban group is not correct, please change the sentence.

**Response:**

We apologize for this. Therefore, we changed this sentence “Clinical practice guidelines showed the effect of amiodarone on the increase in area under the curve (AUC) of dabigatran, edoxaban, and rivaroxaban (12%–60%, 40%, and minor effect, respectively), but no pharmacokinetic data are available for the apixaban group [14].” to “Clinical practice guidelines reported the effect of amiodarone on the increase in area under the curve (AUC) of dabigatran, edoxaban and rivaroxaban (12%–60%, 40% and minor effect, respectively), *but limited pharmacokinetic data are available for the apixaban group* [14].” in the introduction section, page 2 (line 79).

\# In literature I found a paper of Chang et al, which described bleeding outcomes of patients using NOACs and amiodarone. This would be an interesting paper to add to the introduction. Chang SH, Chou IJ, Yeh YH, Chiou MJ, Wen MS, Kuo CT, See LC, Kuo CF. Association Between Use of Non-Vitamin K Oral Anticoagulants With and Without Concurrent Medications and Risk of Major Bleeding in Nonvalvular Atrial Fibrillation. JAMA. 2017 Oct 3;318(13):1250-1259. doi: 10.1001/jama.2017.13883. PMID: 28973247.

**Response:**

Thank you for your suggestion. We added this sentence “The data finding from the previous observational studies is controversial, some studies reported that clinical outcomes of amiodarone combined with apixaban did not increase the risk of stroke and major bleeding compared with warfarin while other studies reported the apixaban levels only [15-19].” in the introduction part, page 2 (line 80).

\# In the discussion paragraph the authors cite this and more other papers. Please phrase the introduction differently, so that previous knowledge from literature and the addition/relevancy of your work is more clear.

**Response:** We apologize for this and thank you for your suggestion. We revised the phrase in discussion section as your comments.

**4 Methods:**

\# Page 3, line 20-22. I think you mean that the recommended dose is 5 mg twice daily. For patients with at least two of the noted characteristics the dose is 2.5 mg twice daily. Or is the dosing guideline different for Thailand? Then the results are less applicable for other countries?

**Response:**

In Thailand, the recommended dose of apixaban in patients with AF are 5 mg twice daily if patients have at least two of the following characteristics: age ≥ 80 years, body weight ≤ 60 kg, or serum creatinine (SCr) ≥ 1.5 mg/dL. The recommended dose is 2.5 mg apixaban orally twice daily based on clinical practice guideline and package insert the same as other countries. However, a pharmacokinetic study in Asian population showed similar levels with the Caucasian population (doi:10.1002/psp4.12347). Therefore, the results of study in Thai population might be applicable for Southeast Asia countries.

\# How was the anti Xa level measured?

**Response:**

Thank you for your comment. This sentence “The anti-Xa levels were measured by the same method as the apixaban levels, using indirect method to standardize in the LMWH scale.” were inserted in material and methods on page 4 (line 10-16).

\# What was used as definition for minor bleeding?

**Response:**

Thank you for your comments. The definition of minor bleeding is “any overt bleeding requiring a medical intervention (hospitalization, surgery or interventional procedure, further diagnostic imaging, laboratory test, or specialist evaluation) and/or treatment discontinuation, and not meeting any of the criteria for major bleeding”. We have change “minor bleeding” to “clinically relevant non-major bleeding (CRNMB)” in page 4 (line 57).

\# Page 4 line 73 – page 5 line 77. Please clarify why 62 patients had enrollment denials. Furthermore, not 38% of the patients were excluded, but in total 76 of the 109 patients were excluded (70%). This is a high number, do the authors expect bias?

**Response:**

We apologize for incorrect information. We corrected this sentence “38% of the patients were excluded” to “70% of the patients were excluded.” We agree with you that this is a high number, and we think that may indicate bias. Therefore, we mentioned about small sample size is a limitation in our study. In addition, we changed the sentence “A total of 38% of the patients were excluded because of enrollment denials in 62 patients, CrCl less than 15 mL/min or required hemodialysis in 12 patients, and inability to conduct self-care in 2 patients.” to “*A total of 69.7% of the patients were excluded because the patients denied to sign inform consent because they denied venipuncture and more than half of patients received medication delivery in 62 patients, CrCl less than 15 mL/min or required hemodialysis in 12 patients, and inability to conduct self-care in 2 patients.*” in the results part on page 5 (line 80).

\# A total of 33 patients is a relatively small group to show statistical differences. Was a power analysis done to investigate which number of patients is necessary? Especially, the univariate and multivariate analysis of the bleeding is done with only 7 bleeds. In my view, it is not valid to draw the conclusion that concomitant use of apixaban amiodaron is not associated with more bleeding based on this small number.

**Response:**

We agree with you and thank you for your suggestion. Therefore, we added this sentence “First, this work used a small sample size and did not evaluate the relationship between bleeding outcomes and apixaban plasma levels, including the anti-FXa activity, in the apixaban-with-amiodarone group. Therefore, multicenter or larger sample sizes should be used in future studies.” in limitations of this study.

\# Table 1, please explain what bleeding history means.

**Response:**

We apologize for unclear explanation. Bleeding history is prior bleeding in this study. Therefore, we changed “bleeding history” to “prior bleeding”.

\# Please add the median and IQR of apixaban dose and treatment duration.

**Response:**

Thank you for your suggestion. We provided the data of the median and IQR of apixaban dose in Table 1 on page 7. Additionally, this sentence was provided, “The treatment duration of apixaban was 9 (IQR 15,39) months.” in the result section, page 6.

\# Were the patients still using apixaban and amiodarone when bleeding was observed during the 1-year follow up.

**Response:**

Thank you for your comments. Patients were still using apixaban and amiodarone when bleeding was observed during the 1-year follow up. However, their physician tried to evaluated for other causes of bleeding.

\# Did the dose of amiodarone or apixaban change during the one year follow up? Now the trough and peak levels measured in the beginning are compared with bleeding events later that year.

**Response:**

Thank you for valuable comment. During the one year follow up, the dose of amiodarone was not changed. Therefore, we assumed that doses of amiodarone have an effect on bleeding outcome at 1 year. In addition, we added the sentence “Finally, due to apixaban plasma levels were not measured in bleeding events later. Therefore, as a result apixaban plasma levels in the bleeding group cannot definitively confirmed that they were over the expected range.” in conclusion section, on page 12 (line 99).

Table 2: trough to peak. The time from apixaban administration to peak describes the time to peak better than the time from trough to peak. Or is always directly after the trough the dose administrated?

**Response:**

Thank you for your suggestion. We changed “trough to peak” to “time to peak” as reviewer suggested in Table 2.

**6 Conclusion:**

\# In the conclusion you state the amiodaron affects the apixaban levels. However, in your results you state that only the trough levels are statistically significantly different. Please extend your conclusions so it concludes that higher peak and trough levels were found when amiodaron was used, but that this was statically significant for trough levels in the small sample size.

**Response:**

Thank you for your valuable comments. We changed the sentence in conclusion part to “Current knowledge from this study showed, that amiodarone affected apixaban plasma at the peak and trough levels, especially trough levels were increased statistically significantly. Based on the results of this study, no clinically significant bleeding events occurred in the comparison of the apixaban-monotherapy and apixaban-with-amiodarone groups. However, this study had a small number of patients, so close monitoring of bleeding symptoms in patients who used concomitant apixaban with amiodarone were necessary.” on page 9, line 91.

\# As stated before, the conclusion that no clinically significant bleeding event occurred when comparing the control and the amiodaron group, is too strong for the small sample size.

**Response:**

Thank you for your valuable comments. We changed the sentence in conclusion part to “Current knowledge from this study showed, that amiodarone affected apixaban plasma at the peak and trough levels, especially trough levels were increased statistically significantly. Based on the results of this study, no clinically significant bleeding events occurred in the comparison of the apixaban-monotherapy and apixaban-with-amiodarone groups. However, this study had a small number of patients, so close monitoring of bleeding symptoms in patients who used concomitant apixaban with amiodarone were necessary.” on page 9, line 91.

**Comment # 6** PLOS authors have the option to publish the peer review history of their article ([what does this mean?](https://journals.plos.org/plosone/s/editorial-and-peer-review-process#loc-peer-review-history)). If published, this will include your full peer review and any attached files.

If you choose “no”, your identity will remain anonymous but your review may still be made public.

Do you want your identity to be public for this peer review? For information about this choice, including consent withdrawal, please see our Privacy Policy.

**Reviewer #1:** No

**Reviewer #2:** No

**Yellow highlight for reviewer 1

**Green highlight for reviewer 2

**Light blue highlight for editor.
