# Supplementary material for: Does amiodarone impact on apixaban levels? The effect of amiodarone on apixaban level among Thai patients with non-valvular Atrial Fibrillation
Source: PLoS One. 2024 Jan 19;19(1):e0295511. doi: 10.1371/journal.pone.0295511 (PMC10798445; doi:10.1371/journal.pone.0295511)
Supplement: Attachment — Submitted filename: Responses to Reviewer-PONE-D-23-05377.docx [file pone.0295511.s002.docx]

**Responses to comments for Manuscript ID: PONE-D-23-05377 [Major Revisions] entitled “Does amiodarone impact on apixaban levels? A significant trough concentration of apixaban among patients with non-valvular atrial fibrillation”**

Please include the following items when submitting your revised manuscript:

- A rebuttal letter that responds to each point raised by the academic editor and reviewer(s). You should upload this letter as a separate file labeled 'Response to Reviewers'.
- A marked-up copy of your manuscript that highlights changes made to the original version. You should upload this as a separate file labeled 'Revised Manuscript with Track Changes'.
- An unmarked version of your revised paper without tracked changes. You should upload this as a separate file labeled 'Manuscript'.
- Reviewers' comments:
- Reviewer's Responses to Questions
- Comments to the Author

**1. If the authors have adequately addressed your comments raised in a previous round of review and you feel that this manuscript is now acceptable for publication, you may indicate that here to bypass the “Comments to the Author” section, enter your conflict of interest statement in the “Confidential to Editor” section, and submit your "Accept" recommendation.**

Reviewer #1: (No Response)

Reviewer #2: (No Response)

**2. Is the manuscript technically sound, and do the data support the conclusions?**

The manuscript must describe a technically sound piece of scientific research with data that supports the conclusions. Experiments must have been conducted rigorously, with appropriate controls, replication, and sample sizes. The conclusions must be drawn appropriately based on the data presented.

Reviewer #1: Partly

Reviewer #2: Partly

**3. Has the statistical analysis been performed appropriately and rigorously?**

Reviewer #1: Yes

Reviewer #2: No

**4. Have the authors made all data underlying the findings in their manuscript fully available?**

The PLOS Data policy requires authors to make all data underlying the findings described in their manuscript fully available without restriction, with rare exception (please refer to the Data Availability Statement in the manuscript PDF file). The data should be provided as part of the manuscript or its supporting information, or deposited to a public repository. For example, in addition to summary statistics, the data points behind means, medians and variance measures should be available. If there are restrictions on publicly sharing data—e.g. participant privacy or use of data from a third party—those must be specified.

Reviewer #1: No

Reviewer #2: Yes

**5. Is the manuscript presented in an intelligible fashion and written in standard English?**

PLOS ONE does not copyedit accepted manuscripts, so the language in submitted articles must be clear, correct, and unambiguous. Any typographical or grammatical errors should be corrected at revision, so please note any specific errors here.

Reviewer #1: Yes

Reviewer #2: No

**6. Review Comments to the Author**

Please use the space provided to explain your answers to the questions above. You may also include additional comments for the author, including concerns about dual publication, research ethics, or publication ethics. (Please upload your review as an attachment if it exceeds 20,000 characters)

**Reviewer #1: I have the following comments for the revised manuscript:**

1. According to the revised version, the study presented in the manuscript was a prospective observational study, in which the study population was first included to draw blood samples for measuring apixaban levels, and then they were followed for bleeding events. This suggested the exposure namely concurrent use of amiodarone was not assigned by the authors, instead, based on the actual use of amiodarone before inclusion (as described in the method section).

**Response:**

Thank you for your valuable comments and suggestion. We added this sentence “All patients who were included, were already receiving apixaban with or without amiodarone before enrollment based on the actual use of amiodarone before inclusion” in the Study participants section.

2. According to Figure 1, 33 patients were included for propensity score matching (1:2) by “age, body weight, SCr and CrCl”, and it happened to be 11 patients and 22 patients who were finally matched successfully as the two groups. Also, sex was not mentioned as a covariate for calculating the propensity score, but according to Table 1, the two groups had exactly the same sex distribution. I doubt whether this is indeed possible (given such a small sample size).

Besides, details about how the propensity score was calculated and how the matching was performed were still absent.

**Response**

Thank you for your valuable comments and suggestion. We apologize for this mistake and we rechecked and reanalyzed so that we changed the numbers in detail of patient’s flow (Fig.1). This sentence “All patients were already receiving apixaban with or without amiodarone before enrollment. A total of 69.7% of the patients were excluded because the patients denied to sign informed consent, because they denied venipuncture and more than half of the patients received medication delivery in 62 patients, CrCl less than 15 mL/min or required hemodialysis in 12 patients, and inability to conduct self-care in 2 patients.” **changed to** “*All patients were already receiving apixaban with or without amiodarone before enrollment. A total of 41 of the patients were excluded because the patients denied to sign informed consent, because they denied venipuncture in 27 patients, CrCl less than 15 mL/min or required hemodialysis in 12 patients, and inability to conduct self-care in 2 patients. Therefore, a total of 68 patients were enrolled. There were 57 and 11 patients receiving apixaban-monotherapy and apixaban with amiodarone groups, respectively. The propensity score matching was used to balance covariates of patients in apixaban-monotherapy group that there were 22 patients in this group. This study classified the patients into two groups: 11 patients were treated with apixaban-with-amiodarone, and 22 were treated with apixaban-monotherapy (control group).”* in result section, page 5 (line 98-109).

The propensity scores were performed to balance covariates in baseline characteristics including age, body weight, SCr and CrCl. After propensity score matching, age, body weight, SCr and CrCl were relatively similar. Due to age and body weight, SCr and CrCl affect to each factor. Therefore, overmatching occurred that can cause nearly the same characteristics between apixaban-monotherapy and apixaban with amiodarone groups. We did not add sex factor in propensity score matching, because there were no clinically meaningful sex-related differences in apixaban levels.

**2. Minor points:**

1) Abstract- Methods: “in patients …” It is better to mention what patients here (i.e., NVAF patients).

**Response:**

Thank you for your suggestion. We changed “This study was a prospective, observational, and single-center research which was conducted from January 2021 to January 2022 *in patients* who received apixaban at a tertiary care hospital located in the center of Bangkok, Thailand.” to “This study was a prospective, observational, and single-center research which was conducted from January 2021 to January 2022 in *NVAF patients* who received apixaban at a tertiary care hospital located in the center of Bangkok, Thailand” in Abstract section.

2) Abstract- Results: “Likewise, Cpeak showed no difference in the non-bleeding (148. 69 [141. 92–283. 45] μg/L) and bleeding (187.74 [1162.92–238.80] μg/L) groups, p=449.” The results are not consistent with Table 4, and there are also several typos, such as “1162.92”, “p=449”

**Response:**

Thank you for your valuable comments and suggestion. We apologize for that mistake. We corrected this sentence Likewise, Cpeak showed no difference in the non-bleeding (148. 69 [141. 92–283. 45] μg/L) and bleeding (187.74 [1162.92–238.80] μg/L) groups, p=449.” to “Likewise, Cpeak showed no difference in the non-bleeding *(187.74 [162.92–239.80] µg/L)and bleeding (148.69 [141.92–283.45] µg/L) groups, p=0.449*.”

3) Abstract- Conclusions: The conclusion is with causal wordings: “… affects…”, “… causes…” With the used study design, the current study could not draw such conclusions.

**Response:**

Thank you for your valuable comments. We changed the sentence “Amiodarone affected the peaks and troughs of apixaban plasma levels. However, it caused no clinically significant bleeding when compared with the apixaban-monotherapy group.” to “Amiodarone *may increase* the peaks and troughs of apixaban plasma levels. However, there were *no differences in bleeding outcomes* when compared with the apixaban-monotherapy group, in Abstract section.

4) Methods: The statistical analysis section still did not cover all the analyses the authors presented in the results.

**Response:**

We apologize for this and thank you for your suggestion. We provided this sentence “A logistic regression analysis was used to determine the factors associated with bleeding.” in Methods section.

5) Results: Median follow-up should be provided in the result section.

**Response:**

Thank you for your suggestion. We provided this sentence “The median (IQR) of follow-up periods were 12 (IQR: 12-12) months” in the result section.

6) Result: “The results showed that the Ctrough and Cpeak were increased by amiodarone.” Since the difference (Table 3) was not statistically significant, I think it is not appropriate to make such a statement.

**Response:**

Thank you for your valuable comments. We changed this sentence “The results showed that the Ctrough and Cpeak were increased by amiodarone.” to “The results showed that amiodarone *may increase* the Ctrough and Cpeak of apixaban.” in result section, page 6 (line 33).

**Reviewer #2:**

Thank you for submitting a new version of the manuscript and the adjustments that are made. Nonetheless, some concerns that were raised in the previous reviewer comments remain and are not (fully) adjusted in this version.

1. You added statements about the small sample size in the discussion. However, in my opinion your sample size it still too small to perform statistical test on the relation between bleeding and the concomitant use of amiodarone with apixaban. You can only use descriptive statistics for the bleeding events that occurred. This makes it also not possible to state there was no significant difference in bleeding between the two groups.

**Response:**

Thank you for your valuable comments. We deleted this sentence “However, no statistically significant difference was observed between apixaban-with-amiodarone and control groups.” in result section, page 7 (lines 56-57).

2. Page 3, line 95. Thank you for adding more relevant literature to the introduction and changing the statement that no pharmacokinetic data was available. Though, while reading this adjusted introduction you get the impression that most studies did not show an increase in risk of stroke and bleeding. While for instance the following two large observational trials showed an increased risk of bleeding when using amiodaron and apixaban. (10.1001/jama.2017.13883, 10.7326/M22-3238)

**Response:**

Thank you for your suggestion. We added this sentence “However, two large observational trials showed an increased risk of bleeding when using amiodarone with apixaban.” in introduction section, page 3 (lines 85-86).

3. You added the apixaban treatment duration of median 9 months. Were patients censored when the stopped using apixaban? Is a bleeding event still relevant for your research question if it occurred after treatment with apixaban?

**Response:**

Thank you for your valuable comments and we apologize that this point was unclear. Censoring of data was performed when occurrence of bleeding or at 1 year. If apixaban discontinued early before 1 year, censoring data was performed. However, all bleeding events occurred during apixaban usage period. Therefore, we provide the sentence “After the blood samples were reported manual electronic chart reviews were performed and all patients were followed until the first occurrence of bleeding outcome or *stopped using apixaban* or until 1 year.” This is in Outcome of interest, on page 4.

4. The English language in the manuscript is improved. However, the manuscript still includes some grammatical errors.

**Response:**

This revised manuscript has been proofread and edited by a native English speaker specializing in Medical English again.

***Other comments, based on the corrections made after input of reviewer 1.***

1. During inclusion of this prospective observational study 33 NVAF patient were enrolled that used apixaban with or without amiodarone. It is correct that from the patients that fulfilled the inclusion criteria (33 patients), exactly 2/3 used apixaban monotherapy and 1/3 used apixaban with amiodaron? This seems like an almost too perfect coincidence?

**Response:**

Thank you for your valuable comments and suggestion. We apologize for this mistake and we rechecked and reanalyzed so that we changed the numbers in detail of patient’s flow (Fig.1). This sentence “All patients were already receiving apixaban with or without amiodarone before enrollment. A total of 41 (37.61%) of the patients were excluded because the patients denied to sign informed consent, because they denied venipuncture in 27 patients, CrCl less than 15 mL/min or required hemodialysis in 12 patients, and inability to conduct self-care in 2 patients. Therefore, a total of 68 patients were enrolled. There were 57 and 11 patients receiving apixaban-monotherapy and apixaban with amiodarone groups, respectively. The propensity score matching was used to balance covariates of patients in apixaban-monotherapy group that there were 22 patients in this group. This study classified the patients into two groups: 11 patients were treated with apixaban-with-amiodarone, and 22 were treated with apixaban-monotherapy (control group).” were changed in result section, page 5 (line 98-109).

2. The authors explain to reviewer 1 that censoring of data was conducted when the bleeding outcome occurred or at 1 year after the blood samples were collected. Bleeding is an event that can occur multiple times, would it not be more appropriate to follow all patients 1 year and take repeated events into account?

**Response:**

Thank you for your valuable comments. We agree with your comments. Therefore, we changed the sentence “After the blood samples were reported manual electronic chart reviews were performed and all patients were followed until the first occurrence of bleeding outcome *or stopped using apixaban* or until 1 year.” in Outcomes of interest section, page 6. However, most patients in this study showed 1 episode of bleeding event, but two patients had more bleeding events.

**Minor:**

#page 4, line 45-47. The SmPC of apixaban states that for NVAF the recommended dose of is 5 mg twice daily. When patients have at least 2 of the following characteristics (age ≥ 80 years, body weight ≤ 60 kg, or serum creatinine (SCr) ≥ 1.5 mg/dL) the dose is 2.5 twice daily. I wonder why you stated exactly the opposite? In the discussion (page 11, line 79) you state the dose correctly. <https://www.ema.europa.eu/en/documents/product-information/eliquis-epar-product-information_en.pdf>

**Response:**

We apologize for this and thank you for your suggestion. This sentence “the standard dose was defined as the administration of 5 mg of apixaban twice daily if patients had at least two of the following characteristics: age ≥ 80 years, body weight ≤ 60 kg, or serum creatinine (SCr) ≥ 1.5 mg/dL. Otherwise, the recommended dose is 2.5 mg apixaban orally twice daily [14].” to “*the standard dose of apixaban is 5 mg twice daily. When patients have at least 2 of the following characteristics: age ≥ 80 years, body weight ≤ 60 kg, or serum creatinine (SCr) ≥ 1.5 mg/dL the recommended dose of apixaban is 2.5 twice daily [14].*” on page 3, lines 22-25.

The recommended dose of apixaban is 2.5 mg taken orally twice daily in patients.

7. PLOS authors have the option to publish the peer review history of their article (what does this mean?). If published, this will include your full peer review and any attached files.

If you choose “no”, your identity will remain anonymous but your review may still be made public.

**Do you want your identity to be public for this peer review? For information about this choice, including consent withdrawal, please see our Privacy Policy.**

Reviewer #1: No

Reviewer #2: No
