# Supplementary material for: Does amiodarone impact on apixaban levels? The effect of amiodarone on apixaban level among Thai patients with non-valvular Atrial Fibrillation
Source: PLoS One. 2024 Jan 19;19(1):e0295511. doi: 10.1371/journal.pone.0295511 (PMC10798445; doi:10.1371/journal.pone.0295511)
Supplement: Attachment — Submitted filename: Responses to Reviewer-PONE-D-23-05377-R3.docx [file pone.0295511.s003.docx]

**Responses to comments for Manuscript ID: PONE-D-23-05377 [Major Revisions] entitled “Does amiodarone impact on apixaban levels? A significant trough concentration of apixaban among patients with non-valvular atrial fibrillation”**

Please include the following items when submitting your revised manuscript:

- A rebuttal letter that responds to each point raised by the academic editor and reviewer(s). You should upload this letter as a separate file labeled 'Response to Reviewers'.
- A marked-up copy of your manuscript that highlights changes made to the original version. You should upload this as a separate file labeled 'Revised Manuscript with Track Changes'.
- An unmarked version of your revised paper without tracked changes. You should upload this as a separate file labeled 'Manuscript'.

**Review Comments to the Author**

**Some comments of the reviewers were not properly addressed. Could you please reconsider these:**

1. You added statements about the small sample size in the discussion. However, in my opinion your sample size it still too small to perform statistical test on the relation between bleeding and the concomitant use of amiodarone with apixaban. You can only use descriptive statistics for the bleeding events that occurred. This makes it also not possible to state there was no significant difference in bleeding between the two groups.'

**Response:**

We would like to thank you for your suggestion. We agree with you in case of too small sample size to analyze the significant factors for bleeding. Finally, we deleted the Table 5 that addresses the univariate and multivariate analyses, as well as the paragraph that mentions about the details of univariate and multivariate analyses.

2. The abstract still contains statements about the bleedings. This statement should be removed. Furthermore, please check the manuscript for statements regarding bleeding (7 bleedings and 2 major bleedings are not high enough numbers to find a significant association or say anything about effects of amiodaron with apixaban versus apixaban monotherapy).

**Response:**

Thank you for your valuable comments and suggestion. We have removed the sentence in the abstract that contains statements about the bleeding following your suggestion. However, we used descriptive statistics for the bleeding events that occurred for explanation, this sentence “*There was* *bleeding which occurred in 7 patients (21.21%); 5 patients in the apixaban-monotherapy group and 2 patients in the apixaban-with-amiodarone group, respectively.*”

In addition, the sentence *“Among the total of 7 patients in the bleeding group; 2 patients received apixaban-with-amiodarone while 5 patients received apixaban-monotherapy”* were provided in Result section, page 6-7.

We deleted the sentence “This study also explored the apixaban plasma levels in 7 patients who had bleeding events. The severity of bleeding was classified as 1) major (2 patients) and 2) CRNMB bleeding (5 patients).” because this sentence is overlapping the previous sentence.

In addition, we rephrased “Based on the results of this study, no clinically significant bleeding events occurred in the comparison of the apixaban-monotherapy and apixaban-with-amiodarone groups.” to “*However, this study could not summarize that amiodarone-with-apixaban was associated with increased bleeding outcomes, compared to apixaban-monotherapy users. Therefore, close monitoring of bleeding symptoms in patients who use concomitant apixaban with amiodarone is necessary*.” in conclusion page 12 (lines 32-35).

3. The answer 'Thank you for your suggestion. We added this sentence “However, two large observational trials showed an increased risk of bleeding when using amiodarone with apixaban.” in introduction section, page 3 (lines 85-86). Observational studies cannot be trials (i.e., if they were trials, they were not observational), please rephrase.

**Response**

Thank you for your suggestion and we apologize for this mistake. We have rephrased “However, two large observational *trials* showed an increased risk of bleeding when using amiodarone with apixaban.” to “However, two large observational *studies* showed an increased risk of bleeding when using amiodarone with apixaban.” in the Introduction section, page 3 (lines 85-86).
